# The reliability of using light therapy compared with LASER in pain reduction of temporomandibular disorders: a randomized controlled trial

**DOI:** 10.1186/s12903-023-02784-8

**Published:** 2023-02-13

**Authors:** Ahmed Fadhel Al-Quisi, Firas A. Jamil, Baseem Natheer Abdulhadi, Salah Jassim Muhsen

**Affiliations:** 1 Department of Oral and Maxillofacial Surgery, Dental Teaching Hospital, College of Dentistry, University of Baghdad, P.O. Box 1417, Bab-Al Moadham, Baghdad, Iraq; 2 Department of Surgery (ENT), College of Medicine, Al-Iraqia University, Baghdad, Iraq

**Keywords:** TMD, Red LED light, LASER, Light therapy

## Abstract

**Background:**

Temporomandibular Disorders (TMD) refer to a group of symptoms where pain is the most leading cause to demand a treatment by the patient. Light therapies are of great importance at current times due to its biosafety and non-invasive quality when used for the management of TMD symptoms. This study aimed to evaluate the efficacy of red LED light with low-level LASER in treating TMD patients.

**Materials and methods:**

A double-blind randomized clinical study was conducted and included 60 patients along 3 groups (20 for each group) presented with myofascial pain related to TMD. Patients were randomly divided into 3 groups. Group A were managed by applying the LED light device into the trigger points without switching the device on. A red LED light was given to group B for 5 min at the tender muscles. Group C were treated by using low-level LASER therapy for 30 s. Patients were evaluated for any improvements regarding the pain score, presence of trigger points, and trismus along 4 visits (1 week interval between each visit). Any side effects related to the 2 devices were also assessed.

**Results:**

Both group B and C patients showed a statistically significant improvement in the pain value (*P* < 0.05) at the 3rd and 4th visits when compared to group A. Regarding tenderness, there was a reduction in the number of trigger points in both study groups; however, the results were insignificant in group B. Statistics showed insignificant differences between group B & C patients regarding pain and number of trigger points at all visits (*P* > 0.05).

**Conclusion:**

Both LED light and LASER therapies could effectively relieve pain associated with myogenic TMD as there were no important differences between their outcomes. However, the biosafety and lower cost of the LED light device compared to the LASER should also be considered.

*Trial Registration* This clinical trial was prospectively registered (TCTR ID: TCTR20190507002) on 07/05/2019. URL: http://www.thaiclinicaltrials.org/show/TCTR20190507002

## Background

Temporomandibular Disorders (TMD) refer to a group of symptoms that may originate from temporomandibular joint (TMJ), muscles of mastication and their associated structures, or both [1–3]. Pain is the most significant feature of the TMD and the most common leading cause for patients to have a treatment [4].

TMD diagnostic criteria have evolved over the last three decades to achieve the most reliable and valid diagnosis. The recent Diagnostic Criteria for TMD (DC/TMD) classify temporomandibular disorders into three groups: Group I: muscle disorders (including myofascial pain with and without mouth opening limitation); Group II: disc displacement with or without reduction and mouth opening limitation; and Group III: arthralgia, arthritis, and arthrosis [5].

TMD is considered the second most common musculoskeletal disorder that causes pain and disability, with an overall prevalence of 90%. Myogenous related TMD are considered the most common type [6, 7]. Patients with myogenic TMD usually complain of pain in the muscles of mastication that are usually aggravated during function, decreased mouth opening, muscle tenderness on palpation, and limitation of mandibular movements [8]. The temporalis and masseter muscles are the main muscles involved in the myogenous type of TMD with irritable trigger points that become painful upon compression. The involved muscles are usually associated with a high concentration of inflammatory mediators that may complicate the situation and result in limitation due to muscle guarding [9]. Despite no clear or specific etiology available to explain the pathophysiology of TMD, it’s well-known that psychological factor like stress may contribute to the development and maintenance of the symptoms of the myogenous related TMD [2, 8].

Different conservative treatment modalities for the arthrogenous type of TMD were implemented. All of them focused on relieving the patient’s pain, which is the crucial cause of disability and financial burden because of the chronic nature of this disease. A recent systematic review has showed a significant decrease in pain intensity after treatment with an occlusal splint and laser therapy [4, 10, 11]. Regarding the myogenous related TMD, radial extracorporeal shock wave therapy combined with physiotherapy was effective in treating the affected masticatory muscles by inducing neovascularization and increasing blood supply, reducing muscle spasm, and producing analgesic effects by blocking the pain signal transmission during treatment [9]. On the other hand, light therapies were proved to be effective in treating TMD with the same treatment principle, which begins as the light being absorbed by the tissue; this absorbed light will transform into heat [12]. The resulted heating will cause vasodilatation of the surrounding blood vessels, increasing blood supply to the affected area with washing out of the inflammatory mediators and relaxation of the smooth muscles, leading to improvement in patient symptoms [2, 12].

These effects of light therapies depend on the level on which their photons being absorbed. LASER can trigger additional photochemical reactions on the mitochondrial level, changing cell metabolism and protein synthesis. Furthermore, it has been suggested that low-level light therapy induces new blood vessels formation with collagen production and increased fibroblast cells activity [13]. These factors besides raised tissue temperature will improve the microcirculation of the irradiated tissue with clearing off most of the inflammatory mediators [4]. The critical difference between these light therapies is the exact wavelength and optical power, which will determine the amount of the delivered energy and the depth of the light penetration through tissue [14].

Despite previous studies had been conducted to evaluate the effectiveness of LASER and red LED light in the management of TMD, no consensus has been reached on the comparable therapeutic abilities of these treatment modalities in relieving myogenous related TMD symptoms that identified by using the recent DC/TMD diagnostic criteria. The current study aims to evaluate the effectiveness of red LED light therapy, compared to low-level LASER, in relieving the symptoms of TMD and the side effects that may exist during treatment. To our knowledge, such comparison with similar parameters has not been studied in the literature.

### Question in focus

Is the light therapy, in comparison with LASER, effective in relieving pain and symptoms of TMD patients?

### Patients and methods

This study was a non-invasive, case–control, randomized and double-blind clinical trial. It has been conducted at the Oral and Maxillofacial Surgery Unit of the Dental Teaching Hospital, from October 2019 to August 2020, according to the ethical principles and in compliance with the Declaration of Helsinki and its later amendments.

**Fig. 1:**
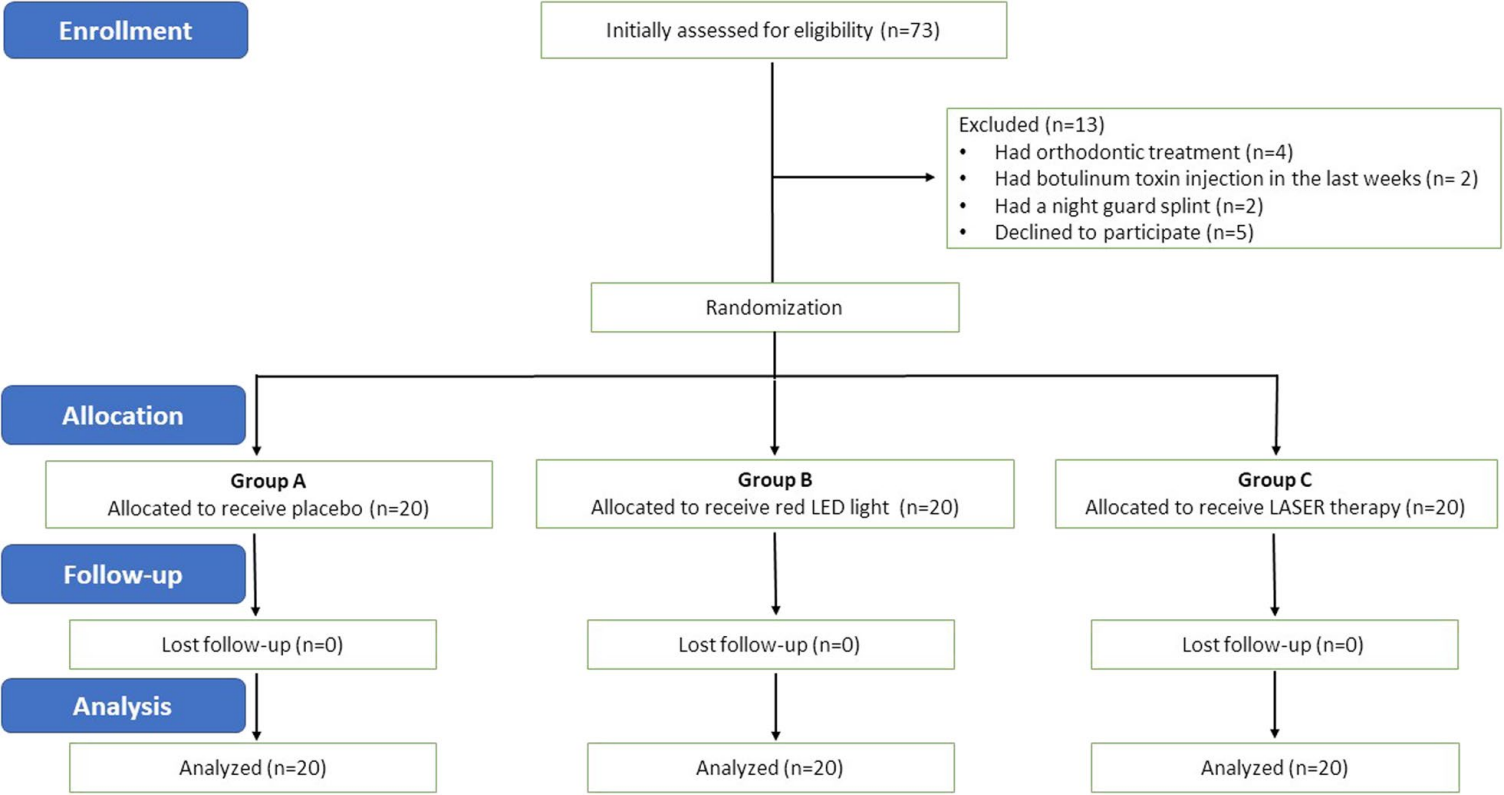
A flow diagram showing patients enrollment along the 3 groups

The included patients were undergraduate students in the College of Dentistry who presented with myofascial pain with or without limitation (Axis Ιa and Ιb) according to the Diagnostic Criteria for Temporomandibular Disorders (DC/TMD) [5]. Any patient who had a history of familiar pain in more than one muscle of mastication with a pain score ≤ 3 on the visual analog scale (VAS) was enrolled in this study. Patients with a history of recent surgical removal of wisdom tooth, orthodontic treatment, TMJ trauma, arthrogenous related TMD, disc displacement (with or without reduction), condylar head fracture, and/or previous treatment of TMD in the last three months were excluded. The study participants’ allocations for different treatment modalities are clearly illustrated in Fig. 1.

Sample size was determined by means of G Power 3.1 software. Informed consent was taken from all participants. Randomization to divide those patients into 3 groups was done by Microsoft Excel 2016, which used to create permuted blocks to ensure that all treatment groups will have the same number of patients. Group A (placebo control group) were managed by applying the red LED light device near the tender points without switching the machine on. Group B (study group) received red LED light to the tender sites that applied for 5 min to each point (masseter, temporalis and pre-auricular muscle areas) extra orally according to the manufacturer instructions. In group C (study group), the patients were treated using low-level LASER therapy applied for 30 s on the affected points (masseter, temporalis and pre-auricular muscle areas) extra orally. The LED light device used in the current study had a wavelength of 660 nm (red light) with a power of 1.6 Watt, which is equivalent to 1.6 J of work per second. The Diode LASER had the following parameters: wavelength = 810 nm, 2.5 Hz, and power = 1 Watt. Additional sessions of LASER were given to group A patients who still complaining from pain related to TMD; all patients were followed up until complete relief of pain.

The initial diagnosis and subsequent evaluations of the treatment progress were made by an independent Maxillofacial Surgeon along 4 visits (1 week interval between each visit); the last one (4th visit) was just a follow-up appointment without any intervention. Any change in the pain score, presence of tenderness in the muscles of mastication (number of trigger points), trismus and/or side effects were assessed and reported.

The treatments for both study groups in the first 3 visits were achieved by other operators. Both participants and the independent maxillofacial surgeon did not know the type of treatment offered to the patients. This clinical trial was prospectively registered (TCTR ID: TCTR20190507002) on 07/05/2019. The study protocol was approved by the Research Ethics Committee (Institutional Review Board).

The statistical analysis was performed using GraphPad Prism version 6.0 for Windows (GraphPad Software, La Jolla, CA, USA). Descriptive statistical analysis included demographic data, calculation of percentage, mean ± standard deviation (SD), and median. Shapiro–Wilk test was utilized to check the normality of data distribution. Mann–Whitney *U-*test was used to compare between non-normally distributed values of two groups. The *P*-value has been considered significant if it was ≤ 0.05.

## Results

Over a period of ten months, 60 dental students along 3 groups (20 in each group) were enrolled in this study; all participants completed the study and no one lost in the follow-up. They were 50 females (83%) and 10 males (17%) whose ages ranged from 19 to 22 years with a mean age of 20.6 years; a detailed demographic features regarding different parameters for all groups are presented in Table 1.

**Table 1:** Demographic features of patients in the 3 groups

Parameter	Group A	Group B	Group C
*Gender (n)*			
Female	14	20	16
Male	6	0	4
Age (mean)	22.6	22.7	21.9
Psychological distress (%)	90	100	85
Duration of symptoms in years (mean)	1.9	2.2	2.1
Previous treatment (%)	20	30	20
Initial pain score^†^ (mean)	3.85	3.8	3.95
Presence of clicking (%)	70	80	90

*n* number of occurrences

^†^By VAS (Visual Analog Scale)

In this study, 92% of the patients gave a history of high stress. In comparison, 8% linked them to the surgical extraction of wisdom teeth. In 45% of the patients, yawning made symptoms worse; while 30% of them found that chewing gum has aggravated the symptoms, and 25% suffered from severe episodes of pain after eating hard food.

According to sites of pain, intraoral application was not made and all interventions by the 2 devices were applied extra-orally on the tender points (masseter, temporalis, and pre-auricular muscle areas). The clinical examination showed a 156 trigger points distributed into the right and left sides of the patient’s faces; TMD affected the left side of the face in 86.7% of the patients.

Statistically, there was a non-significant differences between the mean pain score of the control group (A) and the other 2 study groups (B and C) at the base line visits (*P* = 0.87 and 0.97 for A, B and A, C groups respectively). In the later visits, both group B **(**red LED light**)** and group C (LASER) patients showed a statistically significant improvement in the pain value at the 3rd and 4th (follow-up) visits when compared to group A (placebo) patients (Tables 2 and 3). However, statistics showed no significant differences between group B & C patients concerning pain improvement at all visits (*P* > 0.05) with a *P* value of 0.76 at the last follow-up (fourth) visit.

**Table 2:** Comparison between group A and B patients regarding pain reduction

Periods	Group A (pain score) Mean ± SD	Group B (pain score) Mean ± SD	*P-*value^a^
Base line visit	3.85 ± 1.79	3.8 ± 1.24	0.872^ ns^
2nd visit	3.6 ± 1.15	2.45 ± 1.52	0.091^ ns^
3rd visit	3.55 ± 0.87	2.35 ± 1.66	0.031*
Follow-up visit	3.7 ± 1.79	2.25 ± 1.81	0.017*

^a^By Mann–Whitney *U-*test

^ns^Non-significant

*Significant

**Table 3:** Comparison between group A and C patients regarding pain reduction

Periods	Group A (pain score) Mean ± SD	Group C (pain score) Mean ± SD	*P*-value^a^
Base line visit	3.85 ± 1.79	3.95 ± 2.05	0.976^ ns^
2nd visit	3.6 ± 1.15	3.3 ± 2.54	0.541^ ns^
3rd visit	3.55 ± 0.87	2.3 ± 1.88	0.042*
Follow-up visit	3.7 ± 1.79	2.15 ± 2.16	0.019*

^a^By Mann–Whitney *U-*test

^ns^Non-significant

*Significant

Regarding tender sites, there was a non-significant differences between the mean number of trigger points of the control group (A) and the other 2 study groups (B and C) at the base line visits (*P* = 0.92 and 0.93 for A, B and A, C groups respectively). The results indicated a reduction in the number of the trigger points in group B patients who received red LED light at the 3rd and 4th visits; however, it was statistically insignificant when compared with group A patients (Table 4). On the other hand, there was a highly significant reduction in the number of trigger points in group C patients at the 3rd and 4th visits (Table 5). Again, statistics showed insignificant differences between group B & C patients in reducing trigger points at all visits (*P* > 0.05) with a *P* value of 0.42 at the last follow-up (fourth) visit.

**Table 4:** Comparison between group A and B patients regarding number of trigger points

Periods	Group A (trigger points) Mean ± SD	Group B (trigger points) Mean ± SD	*P*-value^a^
Base line visit	2.75 ± 1.48	2.7 ± 1.59	0.928^ ns^
2nd visit	2.65 ± 1.30	2.3 ± 1.12	0.548^ ns^
3rd visit	2.65 ± 1.13	2.1 ± 1.20	0.177^ ns^
Follow-up visit	2.7 ± 1.38	1.9 ± 1.25	0.06^ ns^

^a^By Mann–Whitney *U-*test

^ns^Non-significant

**Table 5:** Comparison between group A and C patients regarding number of trigger points

Periods	Group A (trigger points) Mean ± SD	Group C (trigger points) Mean ± SD	*P-*value^a^
Base line visit	2.75 ± 1.48	2.7 ± 1.45	0.936^ ns^
2nd visit	2.65 ± 1.30	2.3 ± 1.26	0.322^ ns^
3rd visit	2.65 ± 1.13	1.5 ± 0.88	0.002*
Follow-up visit	2.7 ± 1.38	1.55 ± 0.94	0.004*

^a^By Mann–Whitney *U-*test

^ns^Non-significant

*Significant

There were no complaints about the limitation of mouth opening in all patients and no changes observed during treatment sessions regarding this variable. Both study groups did not report any complication or side effect regarding LED and/or LASER device.

## Discussion

Several previous studies regarding TMD have showed greater frequency and severity of this disease in females than males [2–4, 11]. These findings are consistent with our results, as the patients enrolled were predominantly females. Many factors were assumed to explain these differences, like behavioral, psychosocial, hormonal, and constitutional factors, but no conclusions are drawn [8, 15].

In the current study, results showed that stress was thought to be the leading cause of the TMD symptoms in most patients (92%). This stress could be explained as those students are usually subjected to daily exams in the college together with high educational requirements. Furthermore, the enrolled participants had undergone variable patterns of stress and obstacles through the period of pandemic COVID-19 regarding daily course quizzes, multiple interruptions in their sessions attendance, extended period of final exams (hot weather), and the fear of not completing the academic year.

The left side seemed to be affected (86.7%) more than the right side of the face, which may be related to the right side chewing preferences [16] since unilateral chewing can cause wasting of the masticatory muscles on the left side from hypoactivity, render them more susceptible to injury and myogenous related TMD [17].

Regarding the number of the patients that participated in this study, de Sousa et al. [18] proposed a study protocol designed to compare the effects of phototherapy (infrared LEDs 850 nm, red LED light 660 nm, and placebo control group) on TMD patients diagnosed according to the research diagnostic criteria for temporomandibular disorder—RDC/TMD. The authors suggested that 33 participants (11 patients for each group) will be sufficiently large for a level of significance at *P* = 0.05.

Group A patients have almost showed no any improvement regarding pain nor the number of tender points through all the treatment sessions, although the leading cause of TMD being psychological stress. Placebo and illusional treatment may have no role in patient improvement [19]. Despite this result, the presence of the control group (to compare with) in the TMD studies is highly important since the change in symptoms due to the fluctuating nature of this disease may not reflect the actual achievement of the treatment modality used [20].

In group B patients who treated with red LED light, there was a significant improvement regarding pain scores at the 3rd and 4th visits when compared with placebo group. These findings were in agreement with a previous study conducted by Al-Quisi et al. [2] who specified the useful role of red LED light in the improvement of TMD patients’ symptoms regarding pain, and number of the trigger points. Furthermore, there was a significant improvement in the pain values for group C (LASER) patients at the 3rd and 4th visits compared to placebo group A patients. These outcomes may disagree with Emshoff et al. study [21] who suggested that low-level LASER therapy is not better than placebo in reducing TMJ pain during function. This may be attributed to the use of different type of LASER with different wavelength and power, including patients with arthrogenous TMD who may not respond to light therapy, as well as the limited application of LASER to the pre-auricular area only. At the same time, the results of the current study were in line with recent studies that reported the effectiveness of low-level LASER in reducing pain associated with the myogenous related TMD. [22, 23]

Although both study groups (LED and LASER) showed reduction of the number of the trigger points in comparison with placebo group, the red LED light failed to achieve the same values as the results were insignificant in group B (Table 4). This can be explained by the different mechanism of action for both red LED light and LASER devices. The LED light therapies work on the same basic principles of thermotherapy, as the absorbed light will be transformed into heat and this will increase the cellular nutrition by the increased blood supply to the affected area which in turn leads to increased cell activity and energy production, thus helps in the healing of the tissue and regeneration of new healthy tissue in a phenomenon called photobiostimulation [24–26]. Instead, low-level LASER doesn’t induce thermotherapy but it can stimulate photochemical reaction that changes mitochondrial metabolism, increases ATP production, improving tissue oxygenation by induction for new blood vessels formation, and increases serotonin and endorphin levels [13]. Another cause for this difference in the results regarding tender points might be the short follow-up period.

Nevertheless, the study results have showed that both LED light & LASER had no important differences between their results regarding changes in pain value (improvement) and reduction in the tender sites. These outcomes were consistent with the findings of a recent randomized controlled trial conducted by Herpich et al. [27] who evaluated the short-term combined effects of different types of phototherapy, including LASER (905 nm), infrared (875 nm) and red LED light (640 nm) in TMD female patients (axis I, II, and IIIa) at different doses. The study results revealed a highly significant improvement in pain intensity at the different doses with no reported significant difference between the study groups.

The findings of the current study were in line with a very recent comparative study on TMD patients by Langella et al. [28] who used red LED light (630 ± 10 nm) for one group and low-level laser therapy (850 ± 10 nm) for another group. Treatment was applied for the preauricular, masseter, and temporalis muscle areas for both groups twice a week for four weeks; the pain induced by palpating the masseter muscle and maximum mouth opening was measured by using the Visual Analogue Scale. The authors concluded that both LED and laser therapy showed similar positive results regarding pain intensity and range of mandibular movement in TMD patients.

These previous facts may bring attention to the difference in the cost of the devices, as LED light is much cheaper than LASER device with higher biosafety [29] and lower treatment precautions [30].

## Conclusions

It can be concluded that both red LED light and LASER therapies could effectively relieve symptoms that associated with TMD with no significant differences between their outcomes, but one should also consider both devices’ cost. As well, the biosafety and non-invasive quality of LED light making it the preferred choice over LASER device in the treatment of TMD patients. Within limitations of the study, larger number of participants with longer follow-up periods are recommended.

## Data Availability

The trial protocol, datasets used and/or analyzed during the current study are not publicly available due to privacy and ethical concerns but are available from the corresponding author on reasonable request.

## Abbreviations

TMD: Temporomandibular disorders
TMJ: Temporomandibular joint
VAS: Visual analog scale
SD: Standard deviation
